# Different Serum Free Fatty Acid Profiles in NAFLD Subjects and Healthy Controls after Oral Fat Load

**DOI:** 10.3390/ijms17040479

**Published:** 2016-03-31

**Authors:** Roberto Gambino, Elisabetta Bugianesi, Chiara Rosso, Lavinia Mezzabotta, Silvia Pinach, Natalina Alemanno, Francesca Saba, Maurizio Cassader

**Affiliations:** Department of Medical Sciences, University of Turin, C.so Dogliotti 14, 10126 Torino, Italy; elisabetta.bugianesi@unito.it (E.B.); crosso3@cittadellasalute.to.it (C.R.); lavinia.mezzabotta@unito.it (L.M.); silvia.pinach@unito.it (S.P.); natalina.alemanno@unito.it (N.A.); francescasaba85@yahoo.it (F.S.); maurizio.cassader@unito.it (M.C.)

**Keywords:** nonalcoholic fatty liver disease, free fatty acids, insulin resistance

## Abstract

Background: Free fatty acid (FFA) metabolism can impact on metabolic conditions, such as obesity and nonalcoholic fatty liver disease (NAFLD). This work studied the increase in total FFA shown in NAFLD subjects to possibly characterize which fatty acids significantly accounted for the whole increase. Methods: 21 patients with NAFLD were selected according to specified criteria. The control group consisted of nine healthy subjects. All subjects underwent an oral standard fat load. Triglycerides; cholesterol; FFA; glucose and insulin were measured every 2 h with the determination of fatty acid composition of FFA. Results: higher serum FFA levels in NAFLD subjects are mainly due to levels of oleic, palmitic and linoleic acids at different times. Significant increases were shown for docosahexaenoic acid, linolenic acid, eicosatrienoic acid, and arachidonic acid, although this was just on one occasion. In the postprandial phase, homeostatic model assessment HOMA index positively correlated with the ω3/ω6 ratio in NAFLD patients. Conclusions: the higher serum levels of FFA in NAFLD subjects are mainly due to levels of oleic and palmitic acids which are the most abundant circulating free fatty acids. This is almost exactly corresponded with significant increases in linoleic acid. An imbalance in the n-3/n-6 fatty acids ratio could modulate postprandial responses with more pronounced effects in insulin-resistant subjects, such as NAFLD patients.

## 1. Introduction

Free fatty acid (FFA) metabolism can widely impact on metabolic health. Several metabolic conditions, such as obesity, insulin resistance, type 2 diabetes, and non-alcoholic steatohepatitis, are associated with increased total concentrations of serum free fatty acids [1,2]. Nonalcoholic fatty liver disease (NAFLD) encompasses a spectrum of conditions characterized histologically by hepatic steatosis in individuals without significant alcohol consumption and negative viral, congenital and autoimmune liver disease markers. Hepatic lipid accumulation results from an imbalance between lipid availability and lipid disposal [3,4]. In this context, the composition of serum FFA has been poorly studied so far, especially in the postprandial state [5]. High levels of saturated fatty acids (SFA) were reported to increase coronary risk [6,7].

The liver is the main organ regulating fatty acid metabolism. Several sources supply the liver with a continuous flux of fatty acids [8]. In particular, in the fasting state free fatty acids coming from the lipolysis in adipose tissue fuel the liver. In the fed state, there are two major forms of dietary fatty acids which are available to the liver. In esterified forms, fatty acids are carried to the liver by triacylglycerol–rich chylomicron remnant particles and, as FFA, they stem from the so called spillover mechanism: in the spillover mechanism, FFA are released from chylomicron triacylglycerol by the activity of lipoprotein lipase (LPL, n. EC 3.1.1.34) in peripheral tissues, mainly adipose tissue [9]. Moreover, hepatic “*de novo*” lipogenesis (DNL) from non-lipid precursor increases the content of fatty acid in the liver.

After SFA exposure, *in vitro* experiments had shown that different cell types were induced to synthetize proinflammatory cytokines; they were more prone to apoptosis and had impaired insulin signaling [10,11]. By contrast, the exposure of monounsaturated fatty acids does not seem to trigger apoptosis [12]. Different signaling mechanisms were suggested in order to explain how SFA triggers apoptosis in hepatic cells. Endoplasmic reticulum (ER) stress, mitochondrial dysfunction, Jun N-terminal kinase (JNK) signaling and lipotoxicity are the main molecular mechanisms through which fatty acids exert their deleterious effects on the human metabolism. Wistar rats fed with a diet high in saturated fats showed liver damage and hepatic ER stress [13]. ER stress was due to a decreased fluidity of the lipid bilayer for abnormal incorporation of saturated phospholipids [14]. Excess of unesterified SFA is assembled into saturated phospholipid species leading to stiffening of cellular membranes [15]. Dysregulation of mitochondrial metabolism is due to an imbalance between the glycolytic fluxes and tricarboxylic acid (TCA) cycle since palmitate inhibits glycolytic flux and up regulates TCA cycle and anaplerotic fluxes [16]. The altered mitochondrial metabolism generates an elevated level of reacting oxygen species (ROS) stimulating apoptosis. An accelerated mitochondrial metabolism was observed in NAFLD patients [17]. Additionally, under either ER stress or oxidative stress, molecular signaling arises from JNK activation. Palmitate-induced JNK phosphorylation can be reversed in hepatic cells with administration of antioxidants [18]. SFA shows also a high degree of lipotoxicity. For instance, ceramide synthesis was associated with apoptosis in a hemopoietic precursor cell line [19] and with insulin resistance [20]. In this context, circulating FFAs, which should provide the substrate for triacylglycerol formation, may turn out to be cytotoxic in certain circumstances, such as under insulin resistance. NAFLD is characterized by elevated serum concentration of FFAs, hepatocyte apoptosis, progressive inflammation and fibrosis. In this work, we investigated the composition of circulating FFA in normal and NAFLD subjects during fasting and after a standard oral lipid load. Cultured hepatocytes incubated with FFA of various lengths demonstrated an inverse correlation between FA chain lengths and NAFLD induction [21].

The aim of our work was to study in depth the well-known significant increase in total free fatty acids shown in NAFLD subjects, and to possibly characterize which fatty acids significantly accounted for the whole increase, with specific regard to their classification (saturated, *n*-3, *n*-6 polyunsaturated fatty acids).

## 2. Results

**Figure 1:**
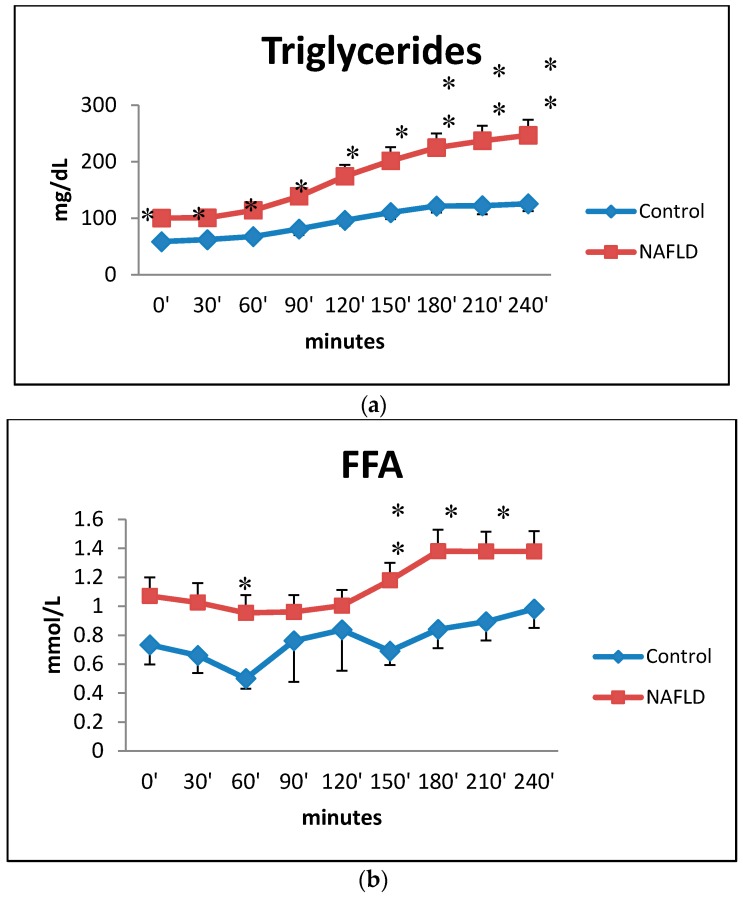
Time courses for total plasma triglycerides (box **a**) and free fatty acids (box **b**) concentrations during the oral fat meal in control (filled diamonds) and nonalcoholic fatty liver disease (NAFLD) (filled squares) subjects. Values are expressed as mean ±SEM. * *p* < 0.05, ** *p* < 0.01.

Main basal features of the patients and control subjects are reported in Table 1. After oral fat load in NAFLD patients, triglycerides reached their maximum peak after around 4 h and they circulated at higher levels than in control subjects. The differences were significant at all times (Figure 1a). The trend of FFA over a 4 h period after the oral fat load is different between NAFLD patients and control subjects (Figure 1b). NAFLD patients showed higher FFA levels than control subjects from baseline through the end of the oral fat load with significant differences at times 60, 150, 180 and 210 min.

**Figure 2:**
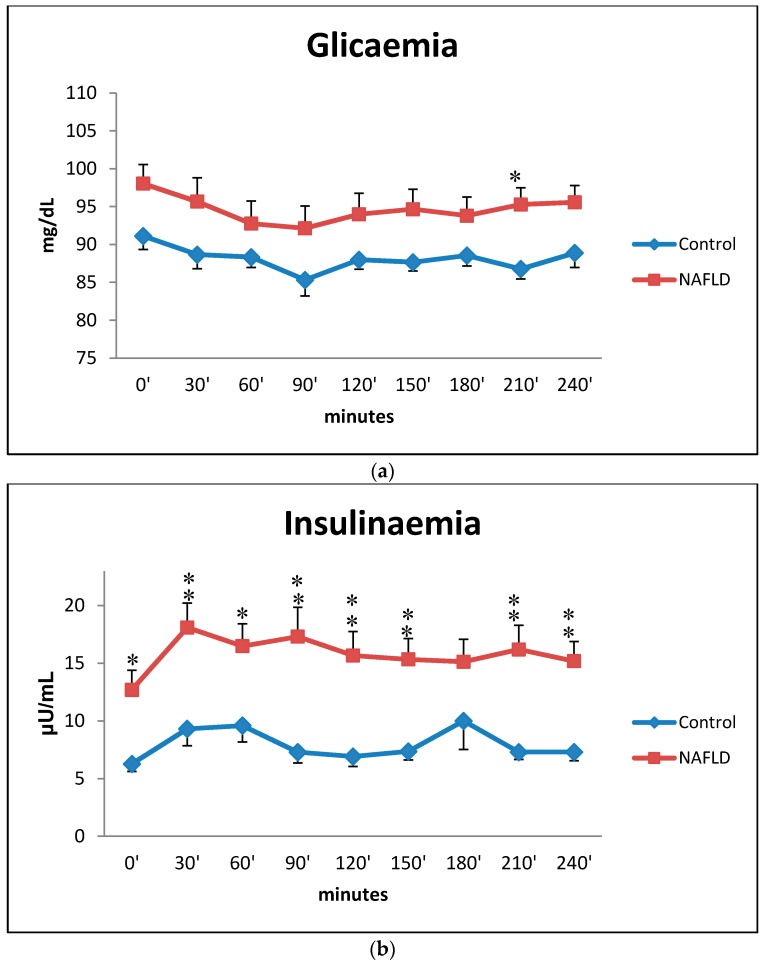
Time courses for glucose (box **a**) and insulin (box **b**) concentrations during the oral fat meal in control (filled diamonds) and NAFLD (filled squares) subjects. Values are expressed as mean ± SEM. * *p* < 0.05, ** *p* < 0.01.

The trend of glycemia is dotted in Figure 2a and it shows a slight decrease from baseline to 90 min both in NAFLD and control group and then a constant course up to 240 min with a statistically significant difference at 210 min. Insulin curve showed in NAFLD patients a major peak at 30 min and higher levels than in control subjects. At all times, except at 180 min, there were statistically significant differences (Figure 2b).

**Figure 3:**
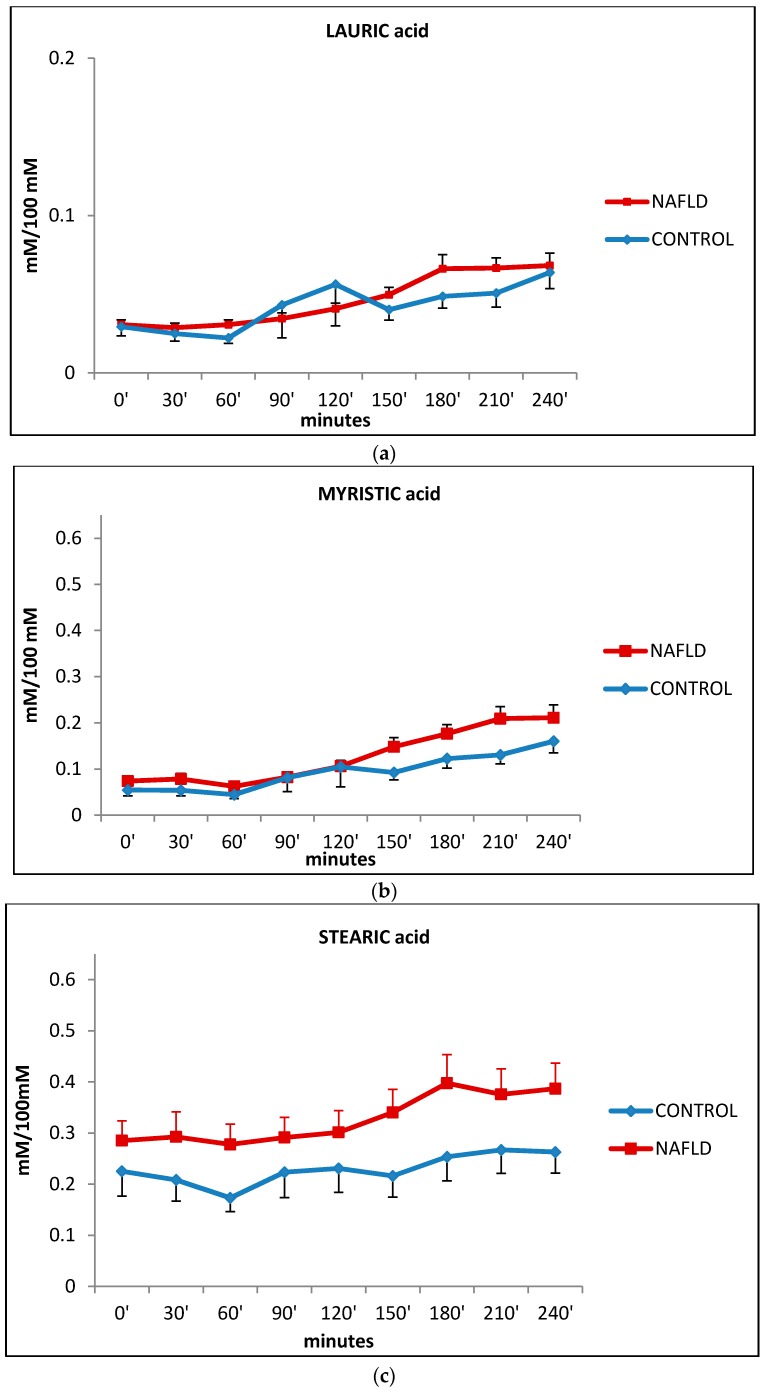
Change in plasma levels of saturated lauric (box **a**), myristic (box **b**) and stearic (box **c**) acid during the oral fat meal in control (filled diamonds) and NAFLD (filled squares) subjects. Values are expressed as mean ± SEM.

Fatty acid values are given as percentage contents (mmol/100 mmol total fatty acids) since the between-individual variations in the molar concentration of total serum FFA is very high [22]. Figure 3 shows the trends of saturated fatty acids lauric (12:0) (a), myristic (14:0) (b) and stearic (18:0) (STA) (c) which did not present significant statistical differences between the two groups.

**Figure 4:**
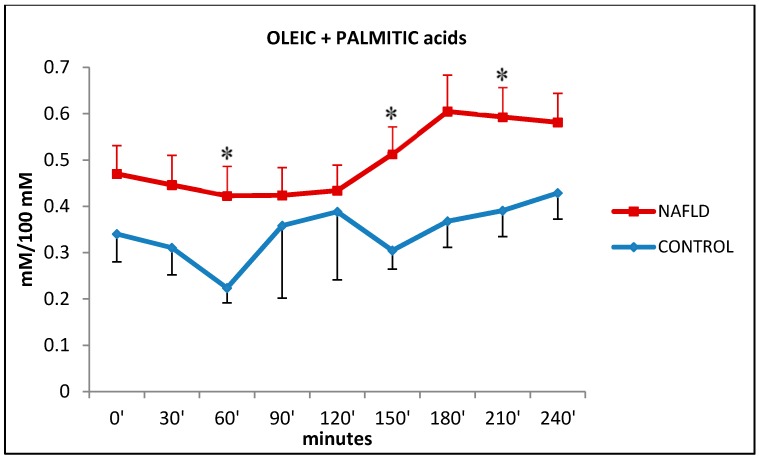
Change in plasma levels of oleic + palmitic acid during the oral fat meal in control (filled diamonds) and NAFLD (filled squares) subjects. Values are expressed as mean ± SEM. * *p* < 0.05.

Figure 4 shows the trends of oleic acid (18:1*n*-9) (OLA) eluted with palmitic acid (16:0) (PAL), a saturated fatty acid. Oleic and palmitic acids amounts reached their peak at 180 min in NAFLD patients and were statistically higher in NAFLD patients than in control subjects at times 60, 150, and 210 min.

**Figure 5:**
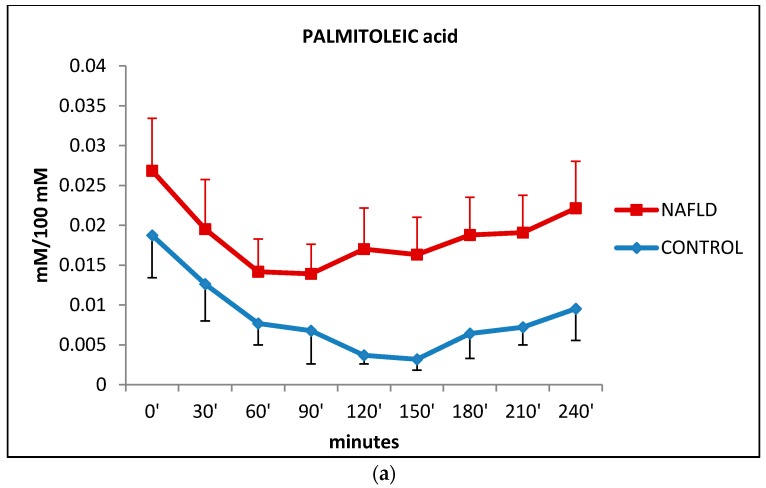

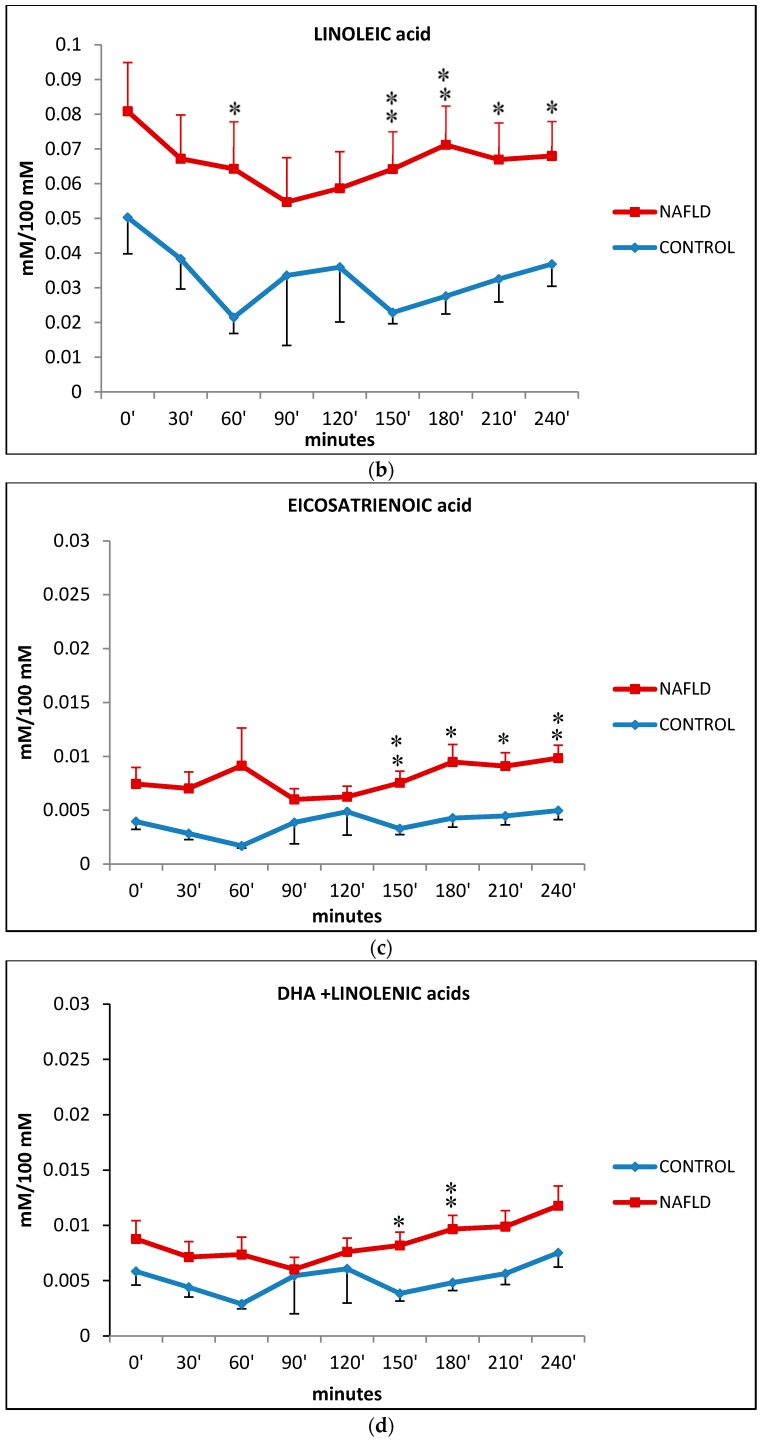

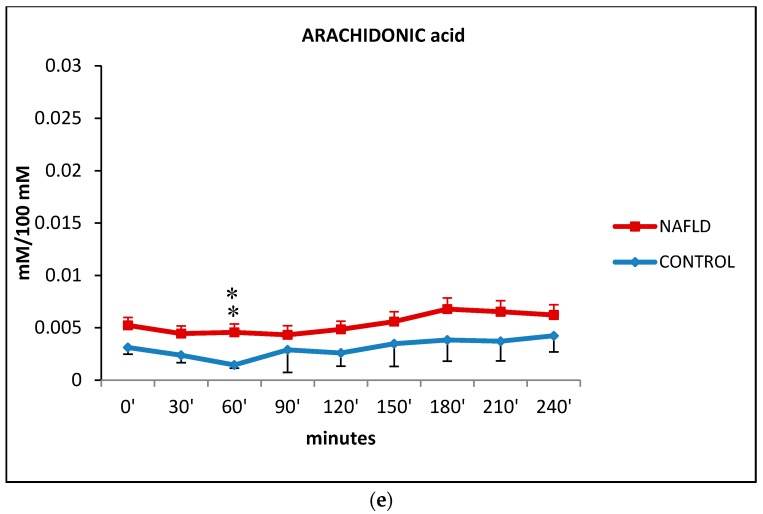
Change in plasma levels of palmitoleic (box **a**), linoleic (box **b**) eicosatrienoic (box **c**), DHA + linolenic (**d**) and arachidonic (**e**) acids during the oral fat meal in control (filled diamonds) and NAFLD (filled squares) subjects. Values are expressed as mean ± SEM. * *p* < 0.05, ** *p* < 0.01.

The monounsaturated palmitoleic acid (16:1*n*-7) fell from baseline to 90 min in NAFLD subjects and from baseline to 150 min in control subjects. Then, in both group palmitoleic acids rose progressively up until the end of the test. No significant differences were observed between NAFLD and control subjects (Figure 5a).

Linoleic acid (18:2*n*-6) (LNA) throughout the fat load showed significant differences at times 60, 150, 180, 210, and 240 min between NAFLD and control subjects (Figure 5b).

Eicosatrienoic acid (20:3*n*-9) in NAFLD patients had higher levels than in control subjects with significant differences from 150 min to the end of the fat oral test (Figure 5c).

Docosahexaenoic (22:6*n*-3) (DHA) and linolenic acids (18:3*n*-3) (ALA), two polyunsaturated fatty acids, are significantly increased in NAFLD patients at 150 and 180 min, compared to control subjects (Figure 5d).

Arachidonic acid (20:4*n*-6) (ARA) showed an almost flat trend in both NAFLD and control group with a significant difference at 60 min (Figure 5e).

**Figure 6:**
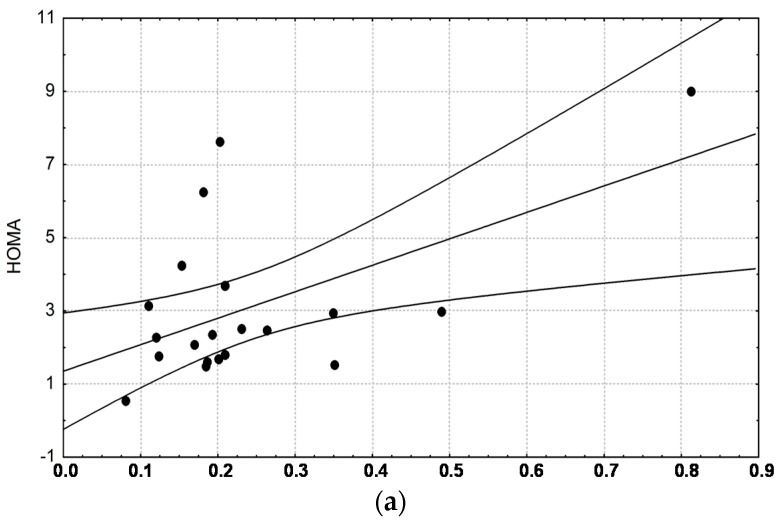

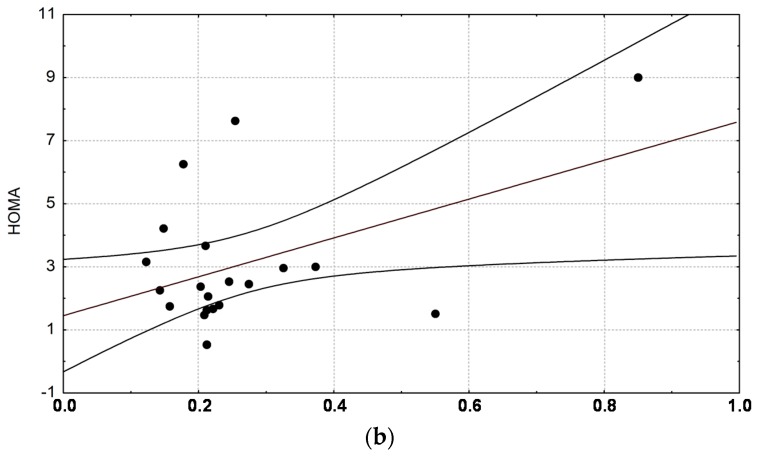
Correlation between homeostatic model assessment HOMA index and n-3/n-6 ratio at time 210 min in NAFLD patients (box 6**a**); correlation between HOMA index and n-3/n-6 ratio at time 240 min in NAFLD patients (box 6**b**); Figure 6a: PUFA n-3/n-6 at 210 min *vs.* HOMA in NAFLD; *r* = 0.54883, PUFA n-3/n-6 at 210 min; Figure 6b: PUFA n-3/n-6 at 240 min *vs.* HOMA in NAFLD; *r* = 0.47351, PUFA n-3/n-6 at 240 min.

The n-3/n-6 ratio measured at every time is not statistically different between control and NAFLD groups. HOMA-IR was higher in NAFLD subjects compared to the control subjects (3.16 ± 2.13 *vs.* 1.42 ± 0.49, *p* = 0.024). HOMA index positively correlated with the n-3/n-6 ratio at time 210 and 240 min in NAFLD patients (*r* = 0.55, *p* = 0.0122 and *r* = 0.47, *p* = 0.035, respectively) (Figure 6a,b).

## 3. Discussion

Free fatty acids derived from the diet can directly enter the circulation through spillover into the plasma FFA pool [23]. Other potential sources of fats causing fatty liver include adipose tissue from where non-esterified fatty acids flow to the liver, *de novo* lipogenesis and through the uptake of intestinally derived chylomicron remnants [24]. After a fatty meal, the FFA profile mirrors that of the meal [25]. We performed an abbreviated 4-hour postprandial fat load which was a valid surrogate for longer oral fat loads [26].

In the postprandial phase, the high insulinaemia and triglyceridemia observed in NAFLD patients confirms the insulin resistance state in these subjects [27]. Insulin does not suppress hormone-sensitive lipase in adipose tissue as in healthy subjects; therefore, in the postprandial phase, adipocyte-derived FFA mix with fatty acids coming from the diet and they can reach the liver.

The peak of triglycerides starts at around 4 h after the fatty meal in NAFLD subjects whilst in control subjects the peak is reached earlier. That means a delayed clearance of triglyceride-rich lipoproteins in NAFLD patients [28].

FFA levels are high in NAFLD patients compared to normal subjects with a trend similar to that of oleic and palmitic acid levels (Figure 1b and Figure 4). This study shows that the higher serum levels of free fatty acids in NAFLD subjects are mainly due to levels of oleic (n9) and palmitic acids (reported as unique value in the data presented) which are the most abundant circulating free fatty acids (60, 150, and 210 min after the oral fat load) (Figure 1b and Figure 4). Almost at the same time, linoleic acid (n6) levels increase significantly (60, 150, 180, 210, 240 min) (Figure 5b). Significant increases were also shown for docosahexaenoic acid (n3), linolenic acid (n-3) (Figure 5d), eicosatrienoic acid (n-9) (Figure 5c), and arachidonic acid (n6) (Figure 5e), although just on one occasion.

High levels of oleic and palmitic acids have molecular implications: oleic acid is the preferred substrate for the synthesis of triglycerides, and cholesteryl esters [29]. Palmitic acid is the substrate for isoforms 1 and 6 of fatty acid elongase (Elov-1 and Elov-6) which are converted into stearic acid [30]. Stearic acid is rapidly converted to oleic acid by the enzyme stearoyl-CoA 9-desaturase (SCD, No. EC 1.14.19.1) in mammalian cells [31]. A single double bond between carbon 9 and 10 is introduced in the chain of palmitic and stearic acids to be converted to palmitoleate and oleate, respectively [24]. The cellular ratio of oleic and stearic acids can affect membrane fluidity and signal transduction leading to an altered composition of membrane phospholipids, triglycerides and cholesterol esters [32]. Subjects exhibiting a hypertriglyceridemic response to a low-fat, high-carbohydrate diet show an increase in the oleic to stearic acids ratio [33].

Eicosatrienoic acid is significantly increased towards the end of the test in NAFLD subjects (Figure 5c). This increase is unlikely to be due to the meal composition since eicosatrienoic acid is of a negligible amount in dairy cream. Rather, eicosatrienoic acid might be derived from oleic acid metabolism [34].

Linolenic and DHA acids are two PUFA n-3 that coelute in our HPLC method. They significantly increase in NAFLD subjects at time 150 and 180 min. Humans have the ability to metabolize linolenic acids to their longer chain DHA even if this conversion is less than 1% in adults [35]. Further studies are needed to verify its physiological functions. The competition between n-3 and n6 fatty acids for the same enzymes and transport systems might explain why linoleic acid showed a flat trend throughout the test, even though they were found at higher concentrations in NAFLD patients than in control subjects.

The n-3/n-6 ratio measured every 30 min is not statistically different even if the ratio is slightly higher in control subjects than in NAFLD patients. When the n-3/n-6 ratio was correlated with HOMA-IR, it was found that HOMA index positively correlated with the n-3/n-6 ratio at times 210 and 240 min only in NAFLD patients (Figure 6a,b). Therefore, subjects who have a basal insulin resistance have higher n-3/n-6 ratio towards the end of the test as if higher n-3 fatty acids could worsen the clearance of triglycerides in NAFLD subjects. On the contrary, in healthy subjects with optimal insulin sensitivity, n-3 fatty acids could have beneficial influence on the lipid clearance. Our data seem to be in contrast with previous studies suggesting that increasing consumption of n-3 PUFA could improve lipid metabolism both in the fasting and postprandial states [36] even if modifying the n-3/n-6 polyunsaturated fatty acid ratio of a high-saturated fat challenge did not acutely change postprandial triglyceride response in men with metabolic syndrome [37]. It is likely for n-3 PUFA to need more than 8 h to exert beneficial effects in subjects with an impaired lipid metabolism [35]. Amount and type of dietary fatty acids can influence postprandial response [38]. Therefore, an imbalance in the n-3/n-6 fatty acids ratio could modulate postprandial response with more pronounced effects in insulin resistant subjects, such as NAFLD patients.

This preliminary study has some limitations due to the reduced number of control subjects available for the study which prevents us from matching by age, sex, BMI and also by physical activity on daily bases. The patients enrolled should be matched for a *de novo* clinical investigation to expand these preliminary results.

## 4. Experimental Section

### 4.1. Subjects

Twenty-one patients (ethics committee of University Hospital San Giovanni Battista of Torino, 00096648, 30 December 2009) with NAFLD (mean age ± SD, 40 ± 9 years, BMI 27.5 ± 3.9 kg/m^2^) attending our Liver Unit were selected according to the following criteria: persistently (at least 12 months) elevated aspartate aminotransferases (AST) and alanine aminotransferases (ALT) in the absence of significant alcohol consumption (defined as <20 in men and <10 g/day in women); ultrasonographic presence of bright liver without any other liver or biliary tract disease. At ultrasounds, the diagnosis of NAFLD was based on four parameters: diffuse hyperechoic echotexture (“bright liver”), increased liver echotexture compared with the kidneys, vascular blurring and deep attenuation. Control subjects had a normal ultrasound liver scan.

Conditions known to be associated with fatty liver were ruled out by the following exclusion criteria: a Body Mass Index (BMI) ≥35 kg/m^2^; positive serum markers of viral, autoimmune or celiac disease; abnormal copper metabolism or thyroid function indices; a diagnosis of diabetes mellitus based on plasma glucose ≥126 mg/dL in fasting conditions or ≥200 mg/dL at +2 h on a standard oral glucose tolerance test, serum total cholesterol ≥220 mg/dL, serum triglycerides ≥160 mg/dL. The patients did not take drugs known to be steatogenic or to affect glucose metabolism and were not exposed to occupational hepatotoxins. The control group consisted of 9 healthy subjects (mean age ± SD, 27 ± 2 years, BMI 21.2 ± 1.6 kg/m^2^) with normal liver enzymes and abdomen ultrasound scan (see Table 1).

### 4.2. Oral Fat Load

NAFLD patients and controls underwent a standard oral fat load to investigate the metabolism of triglyceride-rich lipoproteins and FFAs. The standard fat load consisted of a mixture of dairy cream (38% fat) and egg yolk for a total energy content of 745.22 Kcal. The fat meal was composed of 79.96 g fats, whose 54.32 g was of saturated fatty acids, 21.80 g of monounsaturated fatty acids, 2.82 g of polyunsaturated fatty acids, and 0.45 g of cholesterol. The Table 2 shows the amounts of the most represented fatty acids in the fat meal given to every participant.

The fat load was consumed during a period of 5 min; subjects kept fasting and strenuous activity was forbidden during the test, since exercise can reduce postprandial lipemia. A catheter (Venflon Viggo AB, Helsingborg, Sweden) inserted in the antecubital vein and kept patent during the test was used to draw blood samples at baseline and every 30 min for 4 h for biochemical determinations. Blood samples were collected in tubes containing EDTA as anticoagulant and plasma was immediately frozen. All subjects provided their informed consent for the study, which was conducted in conformance with the Helsinki Declaration.

### 4.3. Biochemical Analyses

Serum glucose was measured by the glucose oxidase method (Sentinel, Milan, Italy) with an intra-assay variation coefficient of 1.07% and an inter-assay variation coefficient of 2.33%.

Triglycerides (Tg) and cholesterol (Chol) were assayed by enzymatic colorimetric assays (Sentinel, Milan, Italy) with an intra-assay variation coefficient of 2.99% and an inter-assay variation coefficient of 3.46% for triglycerides and with an intra-assay variation coefficient of 2.2% and an inter-assay variation coefficient of 3.38% for cholesterol.

HDL-Chol was determined by enzymatic colorimetric assay after precipitation of LDL and VLDL fractions using heparin-MnCl_2_ solution and centrifugation at 4 °C [39], and it had an intra-assay variation coefficient of 2.5% and an inter-assay variation coefficient of 4.1%.

HDL_2_- and HDL_3_-Chol levels were determined according to Gidez *et al.* [40]: HDL_2_ and HDL_3_ lipoproteins were separated after precipitation of Apo B-containing lipoproteins with heparin-MnCl_2,_ and HDL_2_ particles were further precipitated with dextran sulphate. HDL_3_-Chol was determined in the supernatant. HDL_2_-Chol was obtained by subtracting HDL_3_-Chol from total HDL-Chol.

LDL-Chol was measured with a standardized homogeneous enzymatic colorimetric method in order to avoid triglycerides effects on LDL-Chol determination (Sentinel, Milan, Italy).

QUICKI was calculated from fasting glucose and insulin values as previously reported [41].

HOMA was calculated using units of millimoles per liter for glucose and microunits per milliliter for insulin [42].

The determination of fatty acid composition of free fatty acids was performed by high performance liquid chromatography (HPLC) coupled with a fluorescence detector [43]. This procedure enables the analyses of the content and profile of free fatty acids in total lipids extract. For free fatty acids’ analyses, we prepared an acidified sample mixture containing a small volume of serum and 10% acetic acid. The mixture was applied onto C_18_ minicolumn and the column was washed with 10% acetic acid. The fatty acids were eluted with ethyl ether. The ether phase was evaporated and dried under vacuum at room temperature; the residue was dissolved into the derivatization solution containing the labeling fluorescent compound. A very small volume was injected in a HPLC reverse phase column and the free fatty acids profile was obtained within 45 min. Concentrations of each free fatty acid were obtained from a calibration curve made of 10 fatty acids run at 5 levels.

### 4.4. Statistical Analysis

Data were expressed as means ± SD. Between-group comparisons (NAFLD *vs.* control groups) were performed by using independent “*t*-test”. To assess correlations between data, the Pearson correlation coefficient was calculated. Differences were considered statistically significant at *p* < 0.05.

## 5. Conclusions

The postprandial lipid metabolism in NAFLD subjects is very complex and partially understood. Although the excessive flow of FFA from adipose tissue, especially from abdominal obesity (Table 1), to the liver is considered to be the most important trigger of the NAFLD, little is known about the type of free fatty acids reaching the liver. In literature, there are few data dealing with levels of different circulating free fatty acids, but these data were usually measured only at baseline and come from small groups of subjects [5]; however, they confirmed a significant increase of oleic, palmitoleic and palmitic acids at baseline.

Taking into account the results coming out of this study, it would seem advisable for NAFLD subjects to not only follow a saturated fatty acid-free diet, but also be careful not to consume large amounts of n-3/n-6 PUFA. Obviously, these preliminary data should be further confirmed with larger clinical trials which could help to develop tailored nutritional interventions aimed to improving lipid metabolism in NAFLD subjects with the use of dynamic tests.

## Figures and Tables

**Table 1:** Subjects baseline physical characteristics and fasting blood measurements.

Parameters	Control Group (*n* = 9)	NAFLD Group (*n* = 21)	*p*
Age (year)	27 ± 2	40 ± 9	0.001
BMI (kg/m^2^)	21 ± 2	28 ± 4	0.002
Systolic blood pressure (mmHg)	119 ± 4	125 ± 8	0.049
Waist (cm)	73 ± 6	94 ± 8	0.000001
Diastolic blood pressure (mmHg)	80 ± 0	82 ± 9	0.615
Glucose (mg/dL)	91 ± 5	98 ± 11	0.097
Triglycerides (mg/dL)	59 ± 19	100 ± 49	0.021
Cholesterol (mg/dL)	168 ± 27	182 ± 34	0.306
HDL-Chol (mg/dL)	54 ± 13	42 ± 8	0.007
HDL_2_-Chol (mg/dL)	20 ± 8	12 ± 4	0.003
HDL_3_-Chol (mg/dL)	34.22 ± 5	30 ± 5	0.072
LDL-Chol (mg/dL)	107 ± 27	125 ± 29	0.139
FFA (mmol/L)	0.73 ± 0.41	1.07 ± 0.59	0.131
sdLDL (mg/dL)	21 ± 11	31 ± 18	0.127
c-Peptide (pM/mL)	0.54 ± 0.13	0.92 ± 0.35	0.004
Insulin (µU/mL)	6.28 ± 1.99	12.7 ± 7.68	0.021
AST (U/L)	20 ± 4	33 ± 10	0.002
ALT (U/L)	16 ± 4	64 ± 30	0.001
GGT (U/L)	14 ± 12	80 ± 74	0.021
ALP (U/L)	51 ± 21	75 ± 23	0.016
HOMA-IR	1.42 ± 0.49	3.16 ± 2.13	0.024
QUICKI index	0.367 ± 0.02	0.333 ± 0.03	0.008

Abbreviations: HDL-Chol, High density lipoprotein-Cholesterol; HDL_2_-Chol, High density lipoprotein 2-Cholesterol; HDL_3_-Chol, High density lipoprotein 3-Cholesterol; LDL-Chol, Low density lipoprotein-Cholesterol; FFA, free fatty acids; sdLDL, small dense low-density lipoproteins; AST, aspartate aminotransferase; ALT, alanine aminotransferase; GGT, gamma-glutamyltransferase; ALP, alkaline phosphatase; HOMA-IR, homeostatic model assessment-insulin resistance; QUICKI index, quantitative insulin sensitivity check index.

**Table 2:** Fatty acid composition of lipid mixture prepared for the oral fat load.

Fatty Acids	200 g Dairy Cream (38% fat)	NO. 1 Egg Yolk	Total Fat Load
C12:0 (g)	3.02		
C14:0 (g)	9.18	0.013	
C16:0 (g)	21.40	1.020	
C18:0 (g)	7.54	0.630	
Total SFA (g)	52.75	1.67	54.32
C18:1 (g)	18.00	1.30	
Total MUFA (g)	20.48	1.33	21.81
C18:2 (g)	1.52	0.650	
C18:3 (g)	0.20	0.019	
C20:4 (g)		0.120	
Total PUFA (g)	2.07	0.74	2.82
Total fat (g)	75.30	4.66	79.96
Cholesterol (mg)	228.00	213.92	441.92
Proteins (g)	1.60	2.53	4.12
Carbohydrates (g)	2.28		2.28
Kcal	693.20	52.02	745.22
